# Exciton-like electromagnetic excitations in non-ideal microcavity supercrystals

**DOI:** 10.1038/srep06945

**Published:** 2014-11-06

**Authors:** Vladimir Rumyantsev, Stanislav Fedorov, Kostyantyn Gumennyk, Marina Sychanova, Alexey Kavokin

**Affiliations:** 1 Galkin Institute for Physics & Engineering, Donetsk 83114, Ukraine; 2 Mediterranean Institute of Fundamental Physics, 00047 Marino, Rome, Italy; 3 Physics and Astronomy School, University of Southampton, Highfield, Southampton, SO171BJ, United Kingdom

## Abstract

We study localized photonic excitations in a quasi-two-dimensional non-ideal binary microcavity lattice with use of the virtual crystal approximation. The effect of point defects (vacancies) on the excitation spectrum is investigated by numerical modelling. We obtain the dispersion and the energy gap of the electromagnetic excitations which may be considered as Frenkel exciton-like quasiparticles and analyze the dependence of their density of states on the defect concentrations in a microcavity supercrystal.

Photonic structures and metamaterials are presently in the focus of theoretical and experimental interdisciplinary studies, which span laser physics, condensed matter physics, nanotechnology, chemistry and information science^1^,^2^. Many papers have been devoted to realization of light-emitting devices based on polaritonic crystals^3^,^4^. In this context, semiconductor microcavities represent quantum confined optical systems^5^ featured by strong coupling between elementary crystal excitations (excitons) and the optical field. Photonic supercrystals can be built from spatially-periodic systems of coupled microcavities^6^.

The physics of photonic supercrystals is in many ways similar to the physics of crystalline solids. Due to imperfections of the supercrystal lattice photonic gaps may contain impurity states, which are of crucial importance in realistic photonic structures. While the theory of impurity bands and excitons in semiconductor crystals has been developed in 1970–1980s, a similar theory for photonic crystals is yet to be constructed. In this work we carry out a theoretical study of exciton-like electromagnetic excitations in disordered photonic supercrystals composed by coupled microcavities.

Semiconductor microcavities are widely used in optoelectronic devices nowadays^7^,^8^. Nanocavities in photonic crystals^9^,^10^ represent a particular case of microcavities characterized by a discrete photonic spectrum. Nanocavities with embedded quantum dots have been used to demonstrate the strong light-matter coupling regime in Ref. 11 and proposed for realization of quantum solitons coupled to lower-branch polaritons (LBPs)^3^,^4^. Refs. 3, 4 indicate also that chains of microcavities may be used for practical realization of quantum-information processing.

Recent progress in fabrication of reliable semiconductor microcavities with Bragg mirrors and embedded quantum wells led to demonstration of a Bose-Einstein condensation of exciton-polaritons and finding features of their superfluidity^12^,^13^,^14^. In those specified systems polaritons can be treated as a quasi-equilibrium two-dimensional gas of interacting bosonic quasiparticles.

Basing upon the previously developed^3^ concept of photonic structures, Ref. 15 studies a non-ideal polariton supercrystal realized in a system of coupled microcavities, whose atomic subsystem contains impurity clusters. It is important to know the dispersion of electromagnetic eigenmodes in such non-ideal microcavity supercrystals in order to develop opto-electronic and quantum computation devices based on such structures. Here we study dispersions of localized electromagnetic excitations in an array of coupled microcavities, which form a non-ideal supercrystal containing numerous point-like defects.

## Theoretical background

One of the methods of fabrication of polaritonic crystals is the trapping of two-level atoms in an ideal coupled resonator optical waveguide (CROW)^3^ or in a non-ideal photonic structure^15^. Refs. 3, 8, 9, 15 study coupled cavities with dopant atoms. In the present work, we do not consider photon mode coupling with dopant atoms. Instead we concentrate on exciton-like electromagnetic excitations of the disordered multicavity structure. We consider a 2D lattice of microcavities, each characterized by a single confined optical mode. An overlap of optical fields of the eigenmodes of neighboring microcavities is taken into account, so that photons are allowed to move along the surface of the microcavity array. For the sake of generality, we assume that each cell of the photonic supercrystal lattice may contain an arbitrary number of elements.

Hamiltonian *H* of the model system we consider (for more details see also Ref. 3) writes: 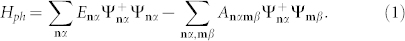
Subscripts **n** and **m** are two-dimensional integer lattice vectors, *α* and *β* numerate sublattices, whose total number is *σ*. *E_nα_*≡ħ*ω***_n_**_*α*_, where *ω***_n_**_*α*_ is the frequency of photonic mode localized in the **n***α*-th site (cavity). Quantity *A***_n_**_*α***m***β*_ defines the overlap of optical fields of the **n***α*-th and **m***β*-th cavities and the transfer of the corresponding excitation, 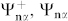 are bosonic creation and annihilation operators describing the photonic mode. Hamiltonian (1) is formally identical to the tight-binding excitonic Hamiltonian in a semiconductor crystal^16^,^17^, for which reason the studied electromagnetic excitations can naturally be referred to as exciton-like. It is worth stressing that we discuss photonic super-crystal excitations and no electronic transitions are involved. Nevertheless, it will be seen below that the dispersion relations of purely electromagnetic crystal excitations in the studied system are quite similar to the Frenkel exciton bands in molecular crystals^16^,^20^.

Let us consider a topologically ordered non-ideal lattice of microcavities with point-like defects, namely vacancies and non-typical microcavities. In such a system, Hamiltonian (1) is no more translation invariant, hence the quantities *ω***_n_**_*α*_and *A***_n_**_*α***m***β*_are configurationally dependent. A convenient tool to study the quasiparticle excitation spectrum in a system with randomly distributed defects consists in configurational averaging of the resolvent of the corresponding Hamiltonian^18^. An averaged resolvent is translation invariant, hence the corresponding elementary excitation spectrum can be characterized by a wave vector **k**. This type of calculation can only be carried out if adopting a certain approximation specific to the considered system. A widespread method of computation of quasiparticle states in disordered media is the virtual crystal approximation (VCA)^18^,^19^. It proves sufficient to elucidate the transformations of elementary excitation spectra under varying defect concentrations. In what follows we rely on this method to compute and analyze the spectrum of electromagnetic excitations as well as the corresponding optical characteristics of the considered non-ideal supercrystal.

Since the VCA consists in replacement of configurationally dependent Hamiltonian parameters with their averaged values, Hamiltonian of a “virtual” crystal 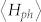 in our case reads as follows: 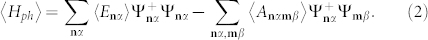
Here angular brackets denote configurational averaging. In an imperfect lattice of coupled cavities quantities *E***_n_**_*α*_ and *A***_n_**_*α***m***β*_ are configurationally dependent and can be written in terms of the random variables 
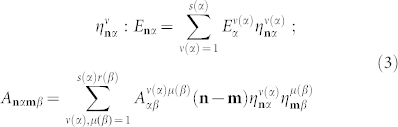
where 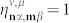 if the **n***α* (**m***β*)-th supercrystal cell is occupied by a *ν*(*α*)-th or *μ*(*β*)-th type of cavity (the total number of types is *s*(*α*) and *r*(*β*) correspondingly) and 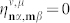 otherwise. Configurational averaging of Eqs. (3) carried out in accordance with the VCA (similarly to the quasiparticle approach^15^,^22^) yields 
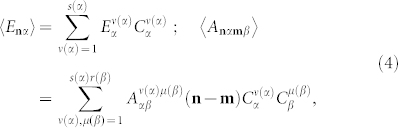
where 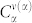
and 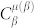 are concentrations of the *ν*-th and *μ*-th types of cavities, 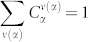. Configurational averaging “restores” the translation invariance of the considered supercrystal system.

Eigenvalues of Hamiltonian (2) are found via its diagonalization by means of the Bogolyubov-Tyablikov transformation^16^,^17^, and are ultimately determined by the system of algebraic equations of the order σ: 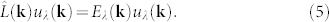
*u_λ_*(**k**) are eigenfunctions of the *σ*×*σ* matrix 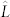 whose elements are expressed through the corresponding characteristics of the Hamiltonian (2): 
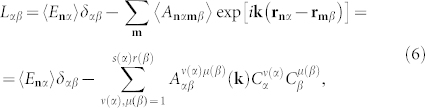
**r_n_**_*α*_being the radius-vector of a resonator belonging to the *α*-th sublattice of the *n*-th elementary cell. The solvability condition of the system (5) 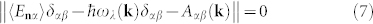
yields the dispersion law *ω_λ_*(**k**) of electromagnetic excitations in the considered photonic supercrystal.

## Results and Discussion

Consider localized electromagnetic excitations in a two-sublattice (*σ* = 2) system of cavities. The left-hand side of Eq. (7) is then a second-order determinant, which when equated to zero gives the following dispersion of photonic excitations: 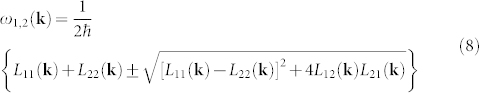
Here *L*_11_(**k**) = *E*_1_−*A*_11_(**k**), *L*_22_(**k**) = *E*_2_−*A*_22_(**k**), *L*_12_(**k**) = −*A*_12_(**k**) and *L*_21_(**k**) = −*A*_21_(**k**) are the matrix elements of operator 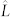.

To be more specific, let us consider a spectrum of electromagnetic excitations in a binary system where each sublattice contains only two types of cavities. In such a case, the quantities 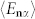 and 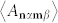 are given by 
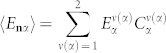
, 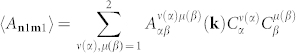.

Being applied to the supercrystal lattice of microcavities where the only defects are vacancies, these expressions take the form 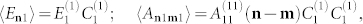

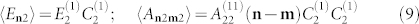

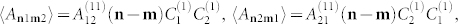


where 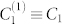 is the cavity concentration in the first sublattice, 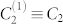 is the cavity concentration in the second sublattice, 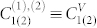 is vacancy concentration in the 1st and/or 2nd sublattices. Concentrations must obviously satisfy the relations 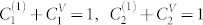. In (9) matrix elements 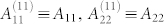, 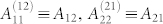, 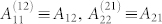 characterize the overlap of optical fields of cavities pertaining to the same sublattice but different cells.

**Figure 1:**
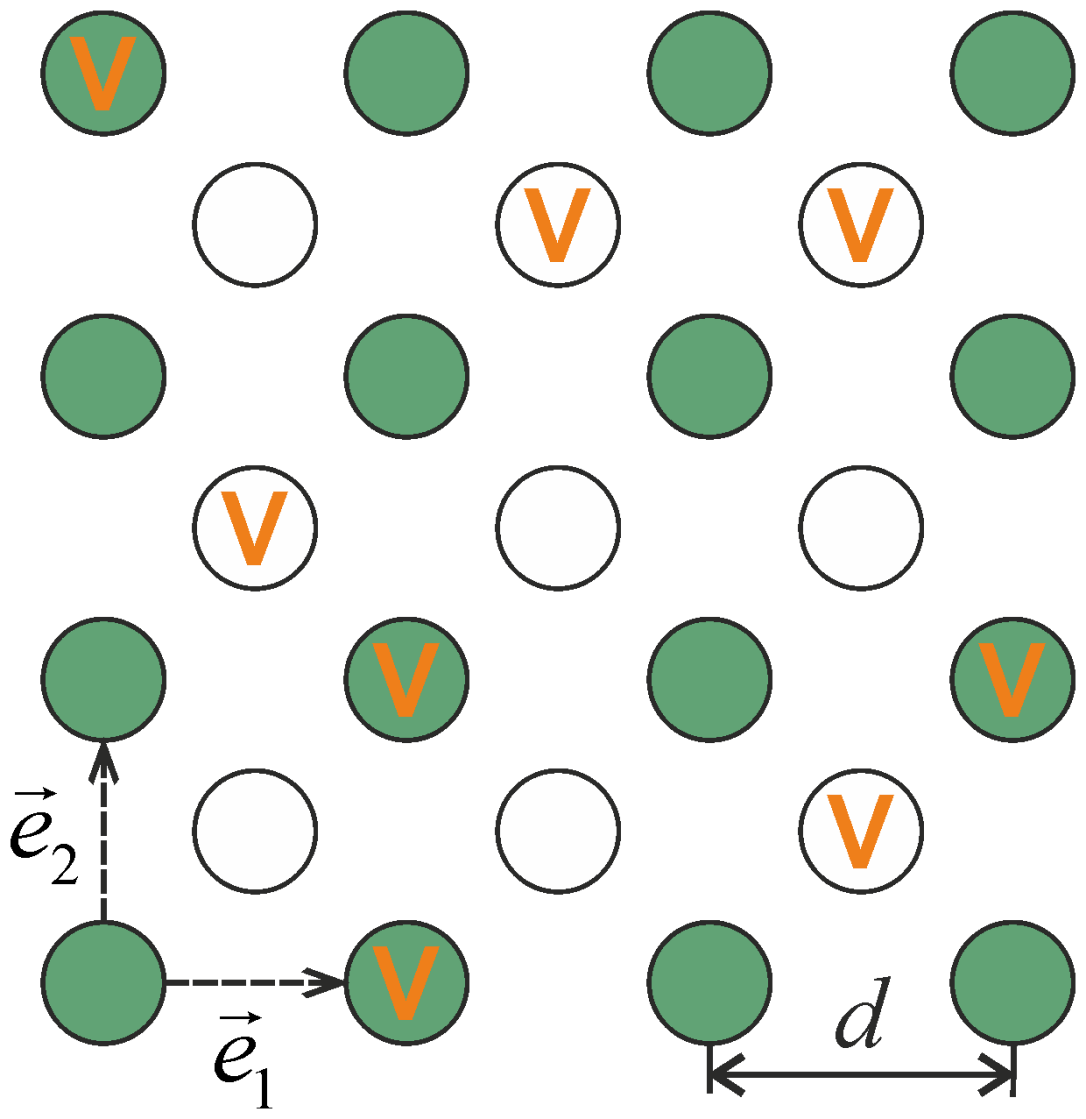
Schematic of a non-ideal two-dimensional two-sublattice system of microcavities, e_1_ and e_2_ are the basis vectors of the square Bravais lattice. “V” denotes vacancies.

The energy spectrum of exciton-like electromagnetic excitations is defined by the type of the considered sublattices and the quantities 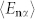 and 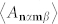. Below we carry out a nearest-neighbor calculation for the case of a square Bravais lattice of period *d*^3^. Location of cavities is defined by the radius-vector **r_n_**_*α*_ = **r_n_**+**r***_α_*, hence their location in the zero elementary cell (**r_n_** = 0) is defined by vectors **r**_01_ = 0 and 
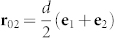
 respectively, where **e**_1_ and **e**_2_ are the basis vectors of the rectangular coordinate system (Fig. 1). In the adopted approximation the matrix elements *A_αβ_*(**k**) can with reasonable accuracy be written as: 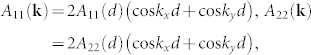


and thus the corresponding matrix elements of operator 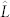 take the form 
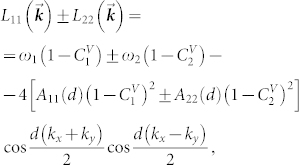

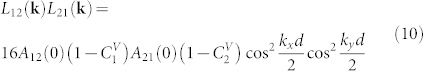


**Figure 2:**
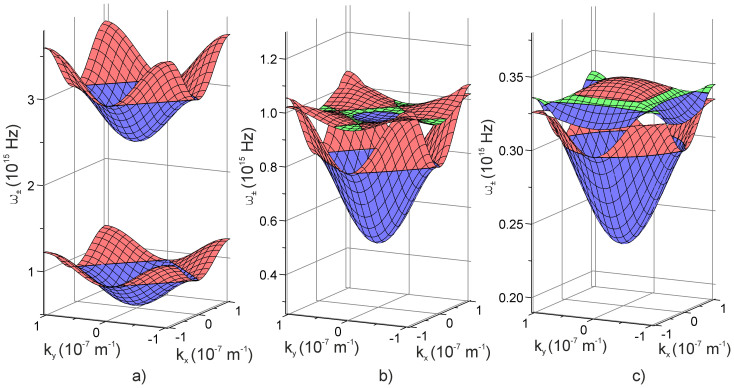
Dispersion 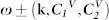 of electromagnetic excitations in the non-ideal two-dimensional two-sublattice system of microcavities for a) 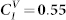, 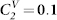; b) 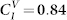, 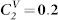, c) 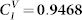, 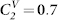.

In (10) the overlap characteristic of optical fields *A*_11(22)_(*d*) defines the transfer probability of electromagnetic excitation between the nearest neighbors in the first (second) sublattice, and *A*_12(21)_(0) is the excitation transfer probability between cavities in the first (second) and second (first) sublattices in an arbitrary cell. Substitution of expressions (10) for *L_αβ_*(**k**) into Eq. (8) gives the dispersion law *ω*_±_(**k**) for electromagnetic excitations (Fig. 2a,b,c). We performed calculation for modeling frequencies of resonance photonic modes in the cavities of the first and second sublattices 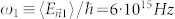
and 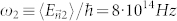 respectively and for the overlap parameters of resonator optical fields 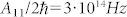, 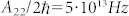 and 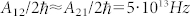. The lattice period was set equal to *d* = 3**·**10^−7^*m*.

**Figure 3:**
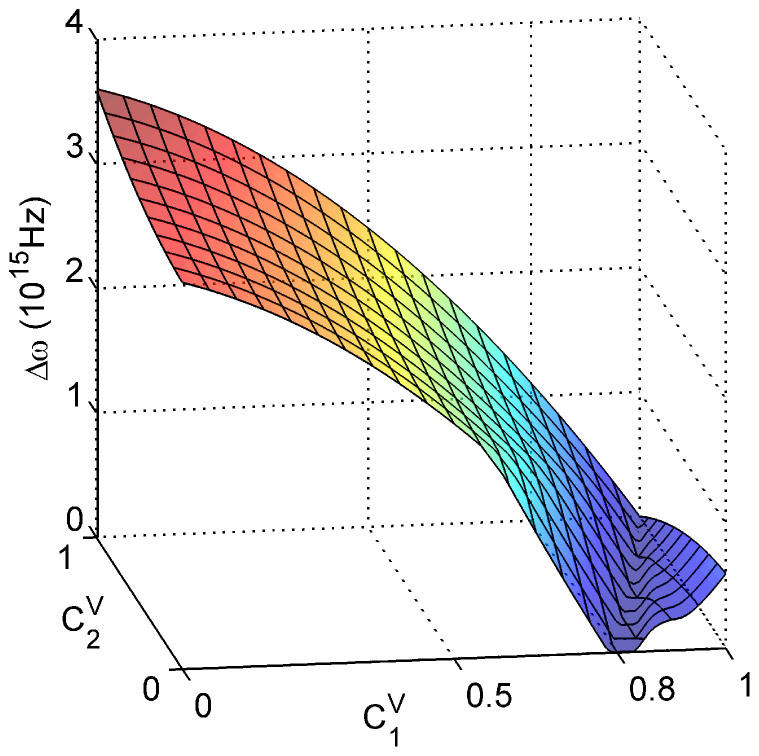
Cavity concentration dependence of the photonic gap width 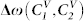 in the studied microcavity supersystem.

Figs. 2a,b,c give three examples of surfaces depicting the dispersion dependence of collective excitation frequencies in the considered non-ideal microcavity lattice. Concentration dependence of the energy gap width 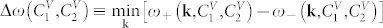 is shown in Fig. 3. The surface 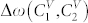 proves non-monotonic and turns to zero in a certain range of 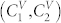. In other words in a certain region of 
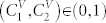
 electromagnetic excitations pass unhindered through the binary two-sublattice microcavity system. Surfaces in Fig. 2a, plotted for 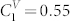, 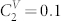 and in Figs 2b,c plotted for 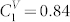, 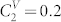 and 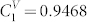, 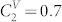 exemplify the cases of 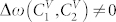 and 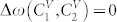 respectively. The presence of two dispersion branches *ω*_±_(**k**) (see Eq. (8)) reflects a two-sublattice structure of the resonator system. For molecular crystals with two molecules in a cell an analogous occurrence of two branches in the dispersion law is referred to as the Davydov splitting of exciton zone^20^.

**Figure 4:**
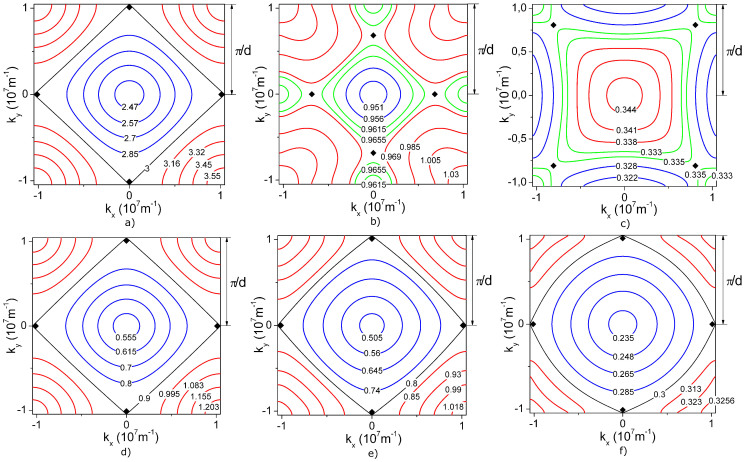
Isofrequency lines for a), d) upper and lower surfaces in Fig. 2a; b), e) upper and lower surfaces in Fig. 2b; c), f) upper and lower surfaces in Fig. 2c. The frequency is measured in the units of 10^15^ Hz. Black diamonds indicate saddle points, which yield singularities in the corresponding densities of states (see Fig. 5).

**Figure 5:**
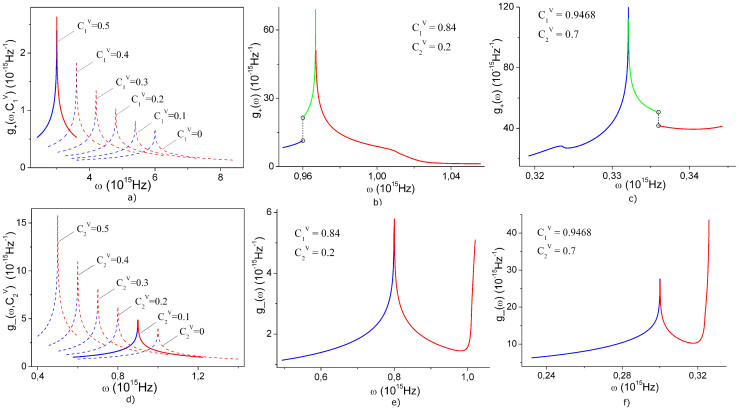
Densities of states for the upper (a) and lower (d) dispersion surfaces 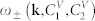 in the range of concentrations 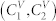, where 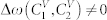 (see Fig. 3). Solid lines correspond to Fig. 2a. Curves a) are valid for any value of 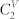
in the range (0…1). Curves d) are valid for any value of 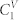 in the range (0…0.8). b) and e) show the densities of states for the upper and lower surfaces, respectively, in Fig. 2b. c) and f) show the densities of states for surfaces in Fig. 2c.

It is important to know how the specificities of the spectrum of the studied quasiparticles are manifested in their density of states 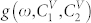. For a non-ideal two-dimensional system with a square lattice the function 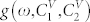 is given by an integral (see Ref. 21): 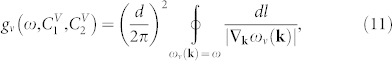
where integration is carried out along an isofrequency contour *ω_ν_*(**k**) = *ω* in the (*k_x_*,*k_y_*)-plane (Figs. 4a,b,c,d,e,f). Dispersion of quasiparticles in the considered system (Figs. 2a,b,c) has nine critical points in the **k**-space (where ∇**_k_***ω*_ν_(**k**) = 0), which is indicative of a possible occurrence of singularities in the density of states 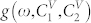. These do in fact always arise as demonstrated in Figs. 5a,b,c,d,e,f. Points 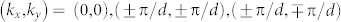 fail to yield a singularity because there the tending to zero gradient ∇**_k_***ω_ν_*(**k**) is offset by a shrinking integration contour, which ultimately gives a finite result of integration in (11) (Figs. 4a,b,c,d,e,f). Singularities of 
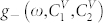
 for the low-frequency dispersion branch are due to the critical points (*k_x_*,*k_y_*) = (0,±*π*/*d*),(±*π*/*d*,0) (indicated by black diamonds in Figs. 4d,e,f). The high-frequency branch in its turn can behave in a more peculiar way. In the case Δ*ω*≠0 the singularities of the high-frequency density of states *g*_+ _are due to the already mentioned points(*k_x_*,*k_y_*) = (0,±*π*/*d*),(±*π*/*d*,0) (black diamonds in Fig. 4a). For Δ*ω* = 0 however the corresponding points may fall inside the Brillouin zone on either the centerlines of the (*k_x_*,*k_y_*)-square (Fig. 4b) or on its diagonals (Fig. 4c). In Figs. 5a,d solid curves show the densities of states 
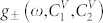
 for the surfaces *ω*_+_ and *ω*_− _in Fig. 2a. Dashed lines show the transformation of functions *g*_+_ and *g*_− _under varying 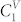 and 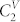. It turns out that in the region 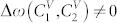 the density of states *g*_+_ is all but independent of 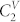, while *g*_− _is almost unaltered by variations in 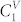. This is explained by the smallness of the term *L*_12_(**k**)*L*_21_(**k**) as compared to [*L*_11_(**k**)−*L*_22_(**k**)]^2^ in Eq. (8). Figs. 5b,c and 5e,f give examples of the typical *g*_+_ and *g*_− _curves for concentration values corresponding to Δ*ω* = 0 (here we took 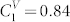, 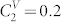 and 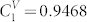, 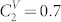). Their evident non-monotonic and discontinuous character is similar to the analogous dependence *g*(*ω*) obtained in Ref. 21 for phonon excitations.

## Conclusion

A number of recent experimental works indicate that microcavity supercrystals may have interesting applications, in particular for creating the optical clockworks of unprecedented accuracy^22^,^23^,^24^. We have used the virtual crystal approximation to model the effect of lattice point defects (vacancies) on the spectrum of exciton-like electromagnetic excitations in a quasi-2D binary microcavity lattice. The energy spectrum of electromagnetic excitations affects the density of states of electromagnetic excitations and alters propagation of normal electromagnetic waves. The obtained dispersions of electromagnetic excitations are noticeably more complex than those of primitive lattices. This complexity is due to the non-ideality of the structure and to the presence of two sublattices. The latter entails multiple manifestations in experimentally observable integral characteristics of optical processes. Evaluation of excitation spectra in more complex photonic systems requires the use of more sophisticated computational methods. Depending on particular cases such can be the one- or multiple-node coherent potential method^18^ and the averaged T-matrix method^25^ along with their various modifications. Our study contributes to the modeling of novel functional materials with controllable propagation of electromagnetic excitations.

## Author Contributions

V.R. proposed the idea and has written the bulk of the paper. V.R., S.F. performed analytical calculations, K.G. and M.S. performed the numerical simulations. V.R., S.F. and A.K. contributed to the discussion and paper writing.
